# Low Levels of Cell-Free Circulating miR-361-3p and miR-625* as Blood-Based Markers for Discriminating Malignant from Benign Lung Tumors

**DOI:** 10.1371/journal.pone.0038248

**Published:** 2012-06-04

**Authors:** Carina Roth, Isabel Stückrath, Klaus Pantel, Jakob R. Izbicki, Michael Tachezy, Heidi Schwarzenbach

**Affiliations:** 1 Department of Tumor Biology, University Medical Center Hamburg-Eppendorf, Hamburg, Germany; 2 General, Visceral and Thoracic Surgery Department and Clinic, University Medical Center Hamburg-Eppendorf, Hamburg, Germany; The Chinese University of Hong Kong, Hong Kong

## Abstract

The high mortality rate of lung cancer patients is mainly due to the late stage at which lung cancer is diagnosed. For effective cancer prevention programs and early diagnosis, better blood-based markers are needed. Hence, blood-based microarray profiling of microRNA (miR) expression was performed in preoperative serum of 21 non-small cell lung cancer (NSCLC) patients and 11 healthy individuals by microfluid biochips containing 1158 different miRs. Two out of the 30 most dysregulated miRs were further validated in serum of 97 NSCLC patients, 20 patients with benign lung diseases and 30 healthy individuals by TaqMan MicroRNA Assays. Microarray profiling showed that miR-361-3p and miR-625* were significantly down-regulated in serum of lung cancer patients. Their further evaluation by quantitative RT-PCR showed that the levels of miR-361-3p and miR-625* were lower in NSCLC than in benign disease (p = 0.0001) and healthy individuals (p = 0.0001, p = 0.0005, respectively). Moreover, the levels of miR-625* were significantly lower in patients with large cell lung cancer (LCLC, p = 0.014) and smoking patients (p = 0.030) than in patients with adenocarcinoma and non-smoking patients, respectively. A rise in the levels of both miRs was observed in the postoperative samples compared with the preoperative levels (p = 0.0001). Functional analyses showed that Smad2 and TGFß1 are not dysregulated by miR-361-3p and miR-625* in the lung cell line A549, respectively. Our present pilot study suggests that miR-361-3p and miR-625* might have a protective influence on the development of NSCLC, and the quantitative assessment of these miRs in blood serum might have diagnostic potential to detect NSCLC, in particular in smokers.

## Introduction

Worldwide, lung cancer is the leading cause of cancer-related death. The carcinoma develops in four major histological types comprising squamous cell lung cancer (SQCLC), adenocarcinoma (ADC) and large cell lung cancer (LCLC) of non-small cell lung cancer (NSCLC), and small cell lung cancer (SCLC) [1]. In the majority of cases the patients develop NSCLC which is associated with an overall 5-year survival of only 15% and a high rate of tumor recurrence [2]. Lung cancer may be caused by exposures to etiologic agents, such as asbestos and cigarette smoke, and by advanced age [3]. Regrettably, not all patients with lung cancer have an operable tumor. The current therapeutic strategies for lung cancer include combinations of cytotoxic chemotherapy and targeted biological therapies, such as bevacizumab and erlotinib [4]. However, adjuvant chemotherapy only results in a modest extension of survival and is significantly toxic for the patients. Furthermore, many patients are not sensitive to treatment due to the resistance of cancer cells. Although tomography-based population screenings for identifying high-risk individuals are carried out, additionally minimally invasive biomarkers allowing repeated blood withdrawals are urgently needed to detect lung cancer early and personalize care of the patients [5].

Since microRNAs (miRs) are known to be detectable as stable molecules in blood of cancer patients [6], they have potential to become new blood-based biomarkers for lung cancer. MiRs are small regulatory, non-coding RNA molecules of approximately 22 nucleotides. They negatively regulate their target mRNA, e.g. the transcripts of tumor suppressor genes, by binding sequence-specifically to complementary sites within the 3′ untranslated regions (UTRs) of mRNAs and inhibit their translation into polypeptides or degrade their target mRNAs [7]. In addition to their role in the repression of translation in proliferating cells, they also mediate activation of translation in G_1_/G_0_ arrest of the cell cycle [8]. This regulation occurs on at least two levels. MiRs seem to be involved in the regulation of different cellular processes, e.g. apoptosis, hematopoietic cell differentiation, metabolism, neural development and metastasis [9], [10], [11]. Since half of human miRs are located in fragile chromosomal regions, which can exhibit amplifications, deletions or translocations, their expression is frequently dysregulated during tumor development [12]. Their expression may be either down- or upregulated in cancer cells as compared to normal cells. Therefore, miRs have tumor-suppressive or oncogenic functions. Specific miRs play critical roles in regulating tumorigenicity. Some miRs may be potential prognostic biomarkers, such as miR-92a-2* in SCLC or miR-155, miR-130a, let-7f and miR-30e-3p in NSCLC, whereas other miRs, such as miR-31, are therapeutic or chemopreventive targets in lung cancer [13]. To improve diagnosis and classification as well as to provide clinical prognostic information in lung cancer, determination of the miR signature in the blood serum of lung cancer patients might be a useful minimally invasive approach.

In the current study we investigated which ones of over thousand known miRs play a role as diagnostic marker in lung cancer, performing a blood-based microarray profiling of miR expression. We further validated two out of the 30 most dysregulated miRs in serum of NSCLC patients in comparison to patients with benign lung disease and healthy individuals by quantitative real-time PCR.

## Materials and Methods

### Ethics Statement

From each participant who was involved in the present study and provided blood samples, written informed consent has been obtained. The use of medical records and blood was approved by the ethics committee of the Medical Board Hamburg (Ethikkommission der Hamburger Ärztekammer, Körperschaft des öffentlichen Rechts, Hamburg, chief executive officer Dr. rer. nat. S. Schrum).

### Patient design and healthy controls

During July 1993 to January 2011, blood serum samples were taken from 118 NSCLC patients before surgery. Due to the limited availability of serum volumes, serum samples from 21 NSCLC patients were used for microRNA profiling, whereas serum samples from 97 NSCLC patients were used for the further validation by quantitative real-time PCR. In 20 cases of these 97 NSCLC patients serial samples were collected approximately two weeks after surgery and before chemotherapy. Serum samples of 20 patients with benign lung disease (9 with benign round foci, 9 with chronic obstructive pulmonary disease, 1 with chronic inflammatory pneumonitis and 1 with pulmonary hypertension) were collected between March 2004 and April 2011 as control cohort. Additionally, 30 healthy controls with no history of cancer and in good health based on self-report were recruited. Table 1 summarizes the parameters of the validation cohorts (97 NSCLC patients, 20 patients with benign lung disease, 30 healthy individuals) those serum samples were analyzed by quantitative real-time PCR. The established risk factors were (e.g. type of carcinoma or smoking behavior) were not available for some patients resulting in divergent numbers of patients in the subgroups listed in table 1.

**Table 1:** Patients' characteristics and correlation of serum RNA and relative levels of circulating miRs with clinical and histopathological parameters of the validation cohort.

Parameters	Patients (%)	Total RNA (ng/µl)	miR361-3p	miR625*
**NSCLC Patients** Mean, Median (95% Confidence Interval)				
Total	97			
Age	65 years			
(range 37–84 years)				
# **Type of carcinoma**				
ADC	39 (45.3)	α **7.4, 6.1 (5.9–8.9)**	9.6, 3.3 (0.3–18.8)	β **1228.1, 806.6 (923.3–1532.9)**
SQCLC	35 (40.7)	α **7.4, 6.7 (6.0–8.7)**	20.8, 4.5 (−3.7–45.4)	β **2535.6, 801.1 (−488.3–5559.6)**
LCLC	12 (14.0)	α **11.8, 8.6 (5.4–18.3)**	6.2, 2.0 (−1.8–14.2)	β **1044.9, 322.5 (−370.1–2459.9)**
**Distant metastasis**				
£M0	52 (89.7)	7.5, 5.7 (6.1–8.9)	12.9, 2.0 (−3.4–29.2)	1983.4, 661.3 (−35.1–4002.0)
$M1	6 (10.3)	9.4, 7.4 (1.9–16.8)	15.7, 3.2 (−12.6–44.1)	748.4, 435.2 (89.6–1407.2)
# **Tumor stage**				
pT1-2	73 (76.8)	7.9, 6.7 (6.6–9.3)	6.3, 3.4 (3.9–8.6)	1112.3, 756.1 (827.9–1396.7)
pT3-4	22 (23.2)	7.8, 5.9 (5.7–9.8)	35.0, 3.2 (−7.1–77.1)	3301.2, 648.4 (−1620.0–8222.3)
# **Lymph node metastasis**				
N0	47 (51.6)	7.9, 6.6 (6.2–9.6)	6.6, 2.7 (3.0–10.3)	1077.2, 582.4 (668.3–1486.1)
N1-3	44 (48.4)	8.1, 6.6 (6.4–9.7)	20.0, 4.0 (−0.7–40.7)	2217.4, 756.1 (−169.6–4604.3)
# **Grading**				
I-II	38 (42.7)	7.2, 5.7 (5.6–8.8)	16.4, 4.0 (−5.9–38.7)	2521.4, 794.6 (−254.7–5297.6)
III-IV	51 (57.3)	8.0, 7.6 (6.9–9.2)	10.4, 2.7 (2.5–18.4)	1029.7, 653.7 (673.5–1386.0)
# **Fumatorium**				
positive	28 (80)	9.7, 7.2 (6.6–12.9)	16.9, 1.6 (−13.8–47.7)	ε **2320.0, 405.4 (−1520.9–6160.8)**
negative	7 (20)	4.9, 5.0 (2.6–7.3)	4.1, 3.4 (0.4–7.8)	ε **1525.1, 1604.0 (500.8–2549.4)**
**Patients with benign lung disease**				
Total	20			
Age	65 years	7.4, 6.3 (5.3–9.4)	47.4, 19.6 (8.9–85.8)	4229.2, 3605.0 (2522.2–5936.2)
(range 37–84 years)				
**Diagnosis**				
benign round foci	9 (45)	6.6, 5.9 (3.8–9.4)	δ **90.2, 57.1 (3.6–184.0)**	4842.6, 3415.8 (400.3–9284.9)
others	11 (55)	7.9, 6.5 (4.7–11.2)	δ **16.2, 14.2 (8.6–23.7)**	3783.1, 3823.8 (2765.6–4800.6)
# **Fumatorium**				
positive	11(55)	8.3, 6.1 (4.8–11.8)	35.5, 24.8 (10.9–60.2)	3094.7, 3226.6 (2082.7–4106.7)
negative	9 (45)	6.0, 6.3 (4.7–7.4)	56.8, 36.0 (29.6–143.2)	5515.3, 3784.2 (1921.7–9109.0)
**Healthy individuals**				
Total	30			
Age	40 years	2.9, 2.7 (2.4–3.4)	27.9, 17.1 (14.2–41.6)	1589.2, 1293.9 (1008.5–2169.9)
(range 21–66 years)				

α p = 0.048,

β p = 0.014,

ε p = 0.030,

δ p = 0.031;

£ M0, patients with localized NSCLC;

$ M1, patients with metastatic NSCLC;

# M0 patients;

p values as determined by Mann Whitney-U test.

### MicroRNA profiling

MicroRNA profiling was performed with serum samples derived from 21 NSCLC patients and 11 healthy individuals. Total RNA was isolated from 1 ml blood serum of 21 NSCLC patients and 11 healthy individuals by Trizol LS Reagent (Karlsruhe, Germany, Invitrogen) according to the manufacturer's protocol. Blood-based microarray profiling was performed on microfluid biochips (Febit Biomed GmbH, Heidelberg, Germany) containing 1158 miRs as published in the current Sanger miRBase release (version 15.0 April 2010 for homo sapiens). For hybridisation of the miRs the Microfluid Primer Extension Assay was used. Specific elongation of bound miRs was carried out by adding Klenow fragment of DNA polymerase I into the channels of microfluid biochips.

### Reference miR

As there is no consensus concerning the normalization of circulating miRs, we evaluated two references (RNU6B and miR-1233) for our miR analyses. MiR-1233 was chosen, because this miR showed the smallest variation between NSCLC patients and healthy individuals as measured by the blood-based microarray. Additionally, RNU6B was chosen, because this miR was recommended by the manufacturer (Applied Biosystems, Darmstadt, Germany). As observed by Huang et al. for colorectal cancer [14], we also measured variable values of RNU6B in our blood serum samples. Therefore, miR-1233 was used for normalization of our miR analyses. We calculated mean values of 12.07 (SD = 2.10), 12.96 (SD = 1.68) and 12.73 (SD = 2.90) for miR-1233 in serum of NSCLC patients, healthy controls and patients with benign lung disease, respectively.

### Quantitative real-time PCR

For isolation of total RNA from 400 µl human blood serum of 97 NSCLC patients, 20 patients with benign lung disease and 30 healthy individuals the mirVana PARIS kit (Ambion, Darmstadt, Germany) was used. Reverse transcription was performed by the TaqMan MicroRNA Reverse Transcription Kit (Applied Biosystems). RNA extraction and cDNA conversion are described elsewhere [15]. The cDNA of all microRNAs was first preamplified in 7.5 µl Taq PCR Mastermix and 0.75 µl TaqMan MicroRNA Assay mix using the Taq PCR Mastermix Kit (Qiagen, Hilden, Germany). The PCR was run on a MJ Research PTC-200 Peltier Thermal Cycler (Global Medical Instrumentation): 1 cycle at 95°C for 5 min, 15 cycles at 95°C for 20 s, 60°C for 20 s and 72°C for 20 s, and a terminal cycle at 72°C for 5 min. For quantitative real-time PCR, the miR-specific TaqMan MicroRNA Assays (Applied Biosystems) for miR-1233 (reference miR), miR-361-3p and miR-625* were used. In a 10 µl-reaction, 3 µl preamplified cDNA were mixed with 5 µl TaqMan Universal PCR Master Mix No AmpErase UNG and 0.5 µl miR-specific TaqMan MicroRNA Assay Mix on a twin-tec real-time PCR plate (Eppendorf, Hamburg, Germany). The quantitative real-time PCR reaction was performed at 95°C for 10 min and for 40 cycles at 95°C for 15 s and 60°C for 60 s on a Mastercycler Realplex (Eppendorf).

The obtained data of the miR expression levels were calculated and evaluated by the ΔCt method as follows: ΔCt = mean value Ct (reference miR-1233) - mean value Ct (miR of interest). The relative expression of miR of interest corresponded to the 2∧(ΔCt)*1000 value.

### Cell culture and transient transfection of miR-361-3p and miR-625*

The lung cancer cell line A549 was cultured in RPMI medium containing 10% FCS (PAA Laboratories, Cölbe, Germany), 2 mMol L-glutamine (Invitrogen) and 200 U/ml penicillin/streptomycin under standard conditions (37°C, 10% CO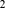).

To analyze whether miR-361-3p and miR-625* have an influence on the expression of Smad2 or the transforming growth factor beta 1 (TGFβ1), 3*10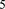 of lung cancer cells were seeded in 6-well plates (NUNC, Roskilde, Denmark) and transfected with the double-stranded miScript miRNA Mimics hsa-miR-361-3p and hsa-miR-625* at final concentrations of 20 µM (Qiagen) and/or with the single-stranded miScript Inhibitors hsa-miR-361-3p and hsa-miR-625* at final concentrations of 200 µM (Qiagen) with 2 µl X-tremeGENE HP DNA Transfection Reagent (Roche Diagnostics, Mannheim, Germany). After incubation of 48 hours, total RNA and protein were extracted using peqGOLD TriFast (Peqlab, Erlangen, Germany) according to the manufacturer's instructions.

### Quantitative real-time PCR and Western Blot of Smad2 and TGFβ1

To determine the mRNA expression of Smad2 and TGFβ1, a total of 200 ng RNA from basal and transfected A549 cells was reverse transcribed using the First strand cDNA synthesis kit (Fermentas, St. Leon-ROT, Germany). The mRNA expression levels were subsequently quantified by real-time PCR using the Maxima SYBR Green/ROX qPCR Master Mix (Fermentas) and the following primers: TGFβ1 forward: 5′-GGCCCTGCCCCTACATTT-3′, and reverse: 5′-CCGGGTTATGCTGGTTGTACA -3′, Smad2 forward: 5′-TACCGAAGGCAGACGGTAACAAGT-3′, and reverse: 5′- GACATGCTTGAGCAACGCACTGAA-3′, and GAPDH forward: 5′- CCCCACACACATGCACTTACC-3′ and reverse: 5′ -CCTAGTCCCAGGGCTTTGATT- 3′.

Protein levels of Smad2 and TGFβ1 in basal and transfected A549 cells were investigated by Western blot analysis. Thirty µg of cell lysates were electrophoretically separated and blotted onto a PVDF membrane (Millipore, Billerica, USA) which was subsequently incubated with antibodies specific for Smad2 (1∶1000, Cell Signaling Technology, Boston MA, USA) and TGFβ1 (1∶1000) overnight. The membrane was reprobed with the anti-Hsc70 antibody (Santa Cruz, Heidelberg, Germany) overnight which served as loading control. Detection of the proteins was carried out using peroxidase-conjugated secondary antibodies (Dako, Glostrup, Denmark) and the chemiluminescence ECL detection Kit (Amersham).

### Statistical analysis

Based on bioinformatic analysis of microRNA profiling, the most dysregulated 30 probes were detected by quotation of mean, median, or variance, parametric t-test, non-parametric Wilcoxon-Mann-Whitney test, empirical Bayes statistics and the area under the receiver operator characteristics (ROC) curves. For significance tests raw p-values were adjusted. To detect possible clusters, hierarchical clustering was carried out.

Two out of the 30 most dysregulated miRs were further validated using the SPSS software package, version 18.0 (SPSS Inc. Chicago, IL). For non-parametric comparisons, univariate analyses of the Mann Whitney-U test of two independent variables, Wilcoxon signed rank test of two dependent variables and bivariate analyses of the Spearman-Rho test were used. Missing data were handled by pairwise deletion. Diagnostic power of the single markers was analyzed by ROC curves. Areas under the curves (AUC) were calculated. A p-value<0.05 was considered as statistically significant. All p-values are two-sided.

## Results

### MiR profiling using a blood-based microarray

**Figure 1:**
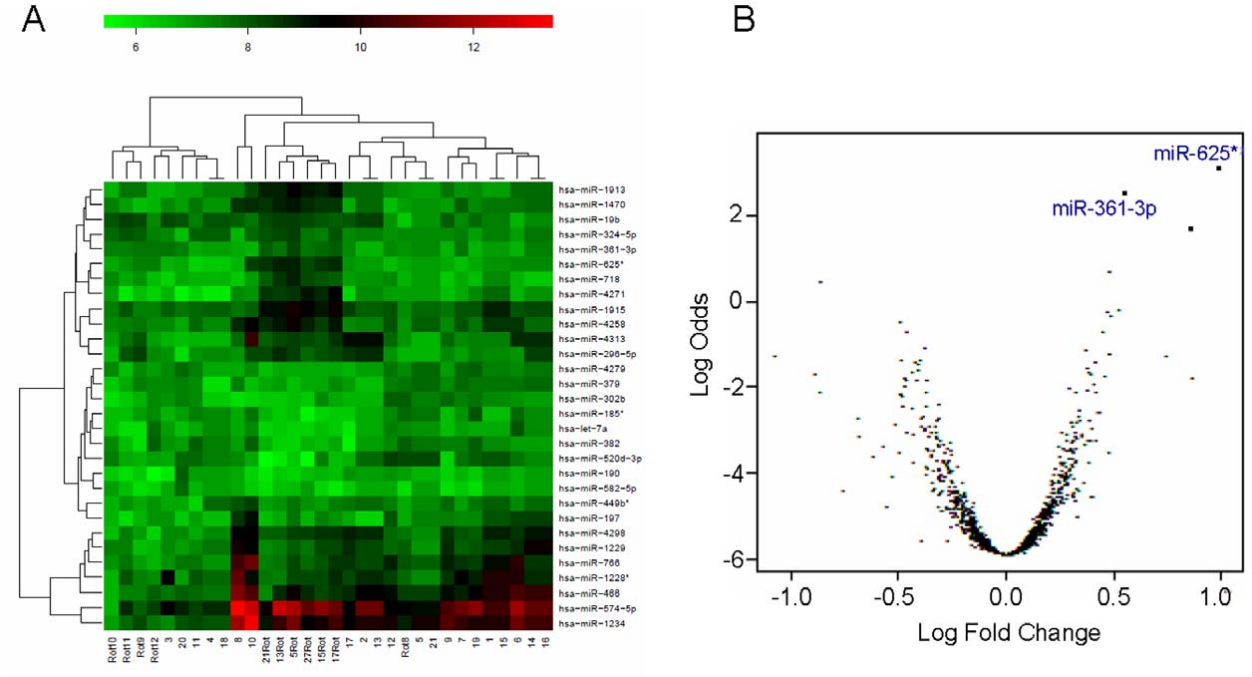
MiR profiling by a blood-based microarray. Hierarchical cluster heat map of miR microarray was performed using microfluid biochips containing 1158 different miRs with serum of 21 NSCLC patients and 11 healthy individuals. The colored representation of samples and probes is ordered by their similarity with a dendogram on top (clustering of samples) and on the right side (clustering of probes) (A). Volcano plot was drawn for comparison of miRs between NSCLC patients and healthy individuals. The two most dysregulated miRs are highlighted in blue on top right (B).

For blood-based miR profiling, microfluid biochips containing 1158 different miRs were used to quantify the expression of miRs in serum of 21 NSCLC patients and 11 healthy individuals. To detect differentially regulated miRs, the quotation of median, paired Student's t-test and limma test were assessed. Dysregulated miRs were detected by the highest absolute value of logarithmized fold changes in comparison of NSCLC patients to healthy individuals. The estimated raw p-values were adjusted for multiple testing, to control the false discovery rate. Table 2 shows 30 most differentially expressed miRs with the adjusted p-values as determined by t-test and limma test. In addition, the normalized median values of healthy individuals and NSCLC patients are listed, and the comparison of these values in both cohorts indicates the down- or up-regulated miRs (Table 2). A similarity matrix was generated containing all pairwise similarities of the serum samples of NSCLC patients and healthy controls. To detect potential clusters in rows (transcripts) and columns (samples) of the normalized expression matrix, hierarchical clustering was carried out. For this analysis 30 miRs with highest overall variability as listed in Table 2 were used (Fig. 1A). In Fig. 1B, the volcano plot shows the comparison of both cohorts (healthy individuals vs. NSCLC patients) and 2 most significantly dysregulated miRs. Based on these array data (Table 2, Fig. 1), we chose miR-361-3p and miR-625*, which were significantly down-regulated in NSCLC patients, for further validation studies.

**Table 2:** Differentially regulated microRNAs in healthy individuals versus patients with NSCLC.

microRNA	normalized median values		t-test adjusted p-value	limma test adjusted p-value
	healthy	NSCLC		
miR-1228	7.73	8.71	0.22	0.12
miR-466	8.25	9.25	0.20	0.12
miR-1229	7.55	8.32	0.19	0.21
miR-4271	8.45	7.00	0.28	0.12
miR-1915	8.98	8.02	0.31	0.25
miR-582-5p	6.30	6.92	0.15	0.12
miR-190	6.37	6.95	0.14	0.12
miR-766	7.66	8.55	0.30	0.14
miR-4313	8.07	7.49	0.62	0.97
miR-1470	8.57	7.58	0.36	0.44
miR-718	7.96	6.83	0.22	0.02
miR-296-5p	8.09	7.28	0.54	0.44
miR-4298	7.89	8.65	0.28	0.34
miR-382	6.66	7.29	0.53	0.23
miR-1234	9.01	9.92	0.56	0.42
miR-19b	8.19	7.62	0.31	0.42
miR-302b	6.61	7.24	0.48	0.37
**miR-361-3p**	**7.65**	**7.09**	**0.06**	**0.01**
miR520-3p	6.71	7.64	0.19	0.06
miR-379	6.88	7.44	0.31	0.52
miR-1913	8.82	7.54	0.23	0.12
miR-449b*	7.11	7.72	0.06	0.14
miR-625*	**8.33**	**7.00**	**0.16**	**0.01**
let-7a	6.40	7.02	0.42	0.14
miR-574-5p	9.65	10.40	0.91	0.76
miR-185*	6.56	7.27	0.30	0.18
miR-4258	8.46	7.77	0.56	0.55
miR-324-5p	8.02	7.46	0.15	0.09
miR-4279	6.89	7.46	0.06	0.12
miR-197	6.82	7.45	0.19	0.25

### Quantification of total RNA, miR-361-3p and miR-625* by qRT-PCR

**Figure 2:**
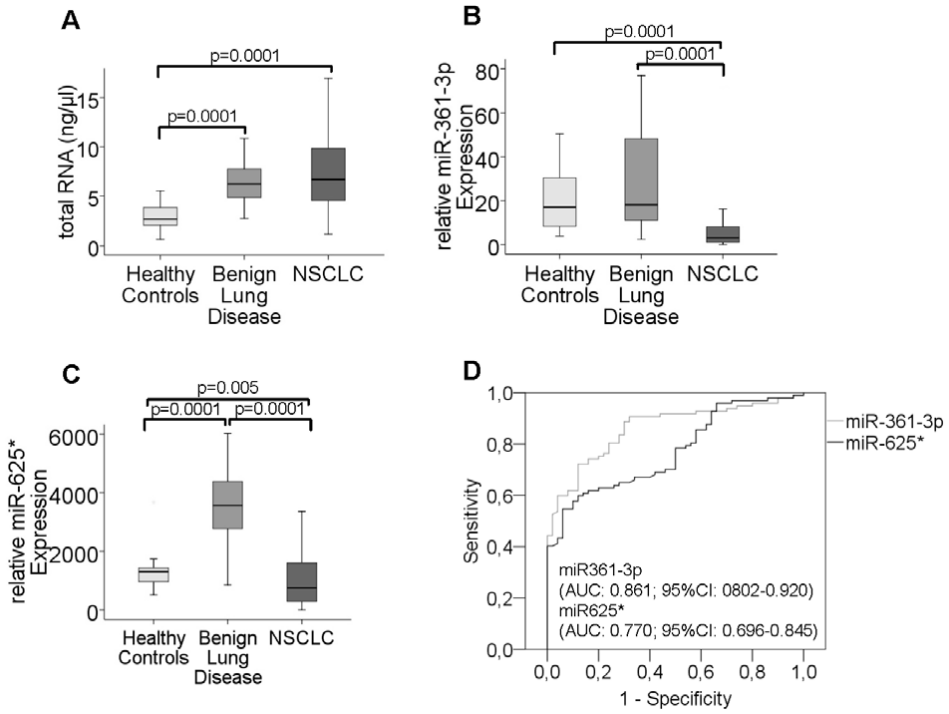
Evaluation of the diagnostic relevance of levels of total RNA and miRs in serum of healthy individuals, patients with benign lung disease and NSCLC patients. The box plots show the different, relative amounts of total RNA (A), miR-361-3p (B) and miR-625* (C) which circulate in blood of healthy individuals (n = 30), patients with benign lung disease (n = 20) and NSCLC patients (n = 97). The relative transcript levels of miRs were determined by the low cycle threshold (Ct) values. As determined by Mann and Whitney-U test, the significant p values of the statistical evaluations of serum RNA and miR levels are indicated. The ROC analysis shows the profile of sensitivity and specificity of miR-361-3p and miR-625* concentrations to discriminate NSCLC patients from patients with benign disease and healthy individuals (D). The AUC values and confidence intervals are indicated.

The concentrations of miR-361-3p and miR-625* were further evaluated in blood serum of 97 NSCLC patients, 20 patients with benign lung disease and 30 healthy individuals. The box plots in Fig. 2 compare the relative levels of total RNA and miRs in healthy individuals and patients with benign or malignant lung disease. Patients with benign (p = 0.0001) and malignant disease (p = 0.0001) displayed significant higher concentrations of total RNA in their blood than healthy individuals (Fig. 2A). In contrast, the levels of miR-361-3p was significantly decreased in NSCLC patients in comparison to healthy individuals (p = 0.0001) and patients with benign lung disease (p = 0.0001), whereas the levels of this miR were similar in healthy individuals and patients with benign lung disease (Fig. 2B). Surprisingly, the relative transcript levels of miR-625* was significant higher in serum of patients with benign lung disease than in healthy individuals (p = 0.0001) and NSCLC patients (p = 0.0001) (Fig. 2C). However, the levels of this miR were significantly lower in NSCLC than in benign disease (p = 0.0001) and healthy individuals (p = 0.0005, Fig. 2C).

In order to determine the sensitivity and specificity of miR-361-3p and miR-625* in distinguishing NSCLC patients from patients with benign disease and healthy individuals, we performed ROC analysis. The AUC values of miR-361-3p and miR-625* were 0.861 and 0.770, respectively, demonstrating the significant difference of the transcript levels between NSCLC patients and the two other cohorts (Fig. 2D). Thus, the yields of miR-361-3p and miR-625* are cancer-specifically decreased in NSCLC patients.

Albeit the cohort of 20 patients with benign lung disease was small, we compared the serum concentrations of miR-361-3p and miR-625* in patients with pulmonary round foci (n = 9) with those in patients with other benign lung diseases (9 chronic obstructive pulmonary disease, 1 chronic inflammatory pneumonitis, 1 pulmonary hypertension). The statistical evaluation of serum concentrations of the miRs in this cohort showed that patients with other benign lung diseases had significantly lower serum levels of miR-361-3p (p = 0.031) than patients with round foci (Table 2).

### Pre- and postoperative serum levels of circulating RNA and miRs in NSCLC patients

**Figure 3:**
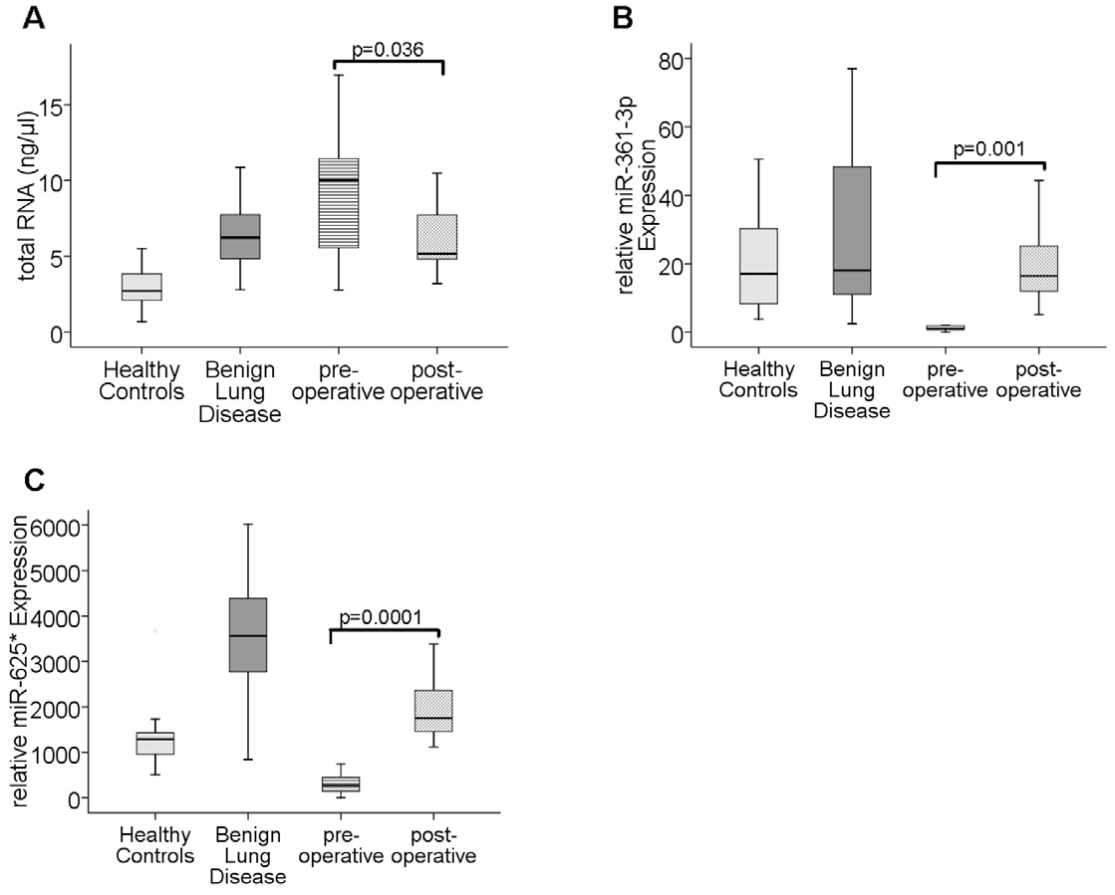
Comparison of total RNA and miR levels in pre- and postoperative serum samples of NSCLC patients. The box plots show the different, relative amounts of total RNA (A), miR-361-3p (B) and miR-625* (C) which circulate in blood of healthy individuals (n = 30), patients with benign lung disease (n = 20) and NSCLC patients (n = 20) collected before and after surgery. As determined by Wilcoxon test, the significant p values of the statistical evaluations of serum RNA and miR levels are indicated above the blots.

In 20 NSCLC patients additional serum samples were collected approximately two weeks after surgery and before the start of any chemotherapy. The high preoperative concentrations of circulating total RNA significantly decreased in postoperative serum samples (p = 0.036, Fig. 3A). In contrast, serum levels of miR-361-3p (p = 0.001, Fig. 3B) and miR-625* (p = 0.0001, Fig. 3C) significantly increased after surgery. The postoperative levels of RNA and miRs displayed similar levels to those of healthy individuals or patients with benign lung disease.

### Decreased serum levels of miR-625* in patients with LCLC and smoking patients

We performed statistical analyses of the preoperative concentrations of circulating total RNA, miR-361-3p and miR-625* with the clinical and histopathological data of the 97 NSCLC patients, included the smoking behavior. Whereas all non-smoker had ADC, the smoker subgroup had different histological types of ADC, SQCLC and LCLC.

**Figure 4:**
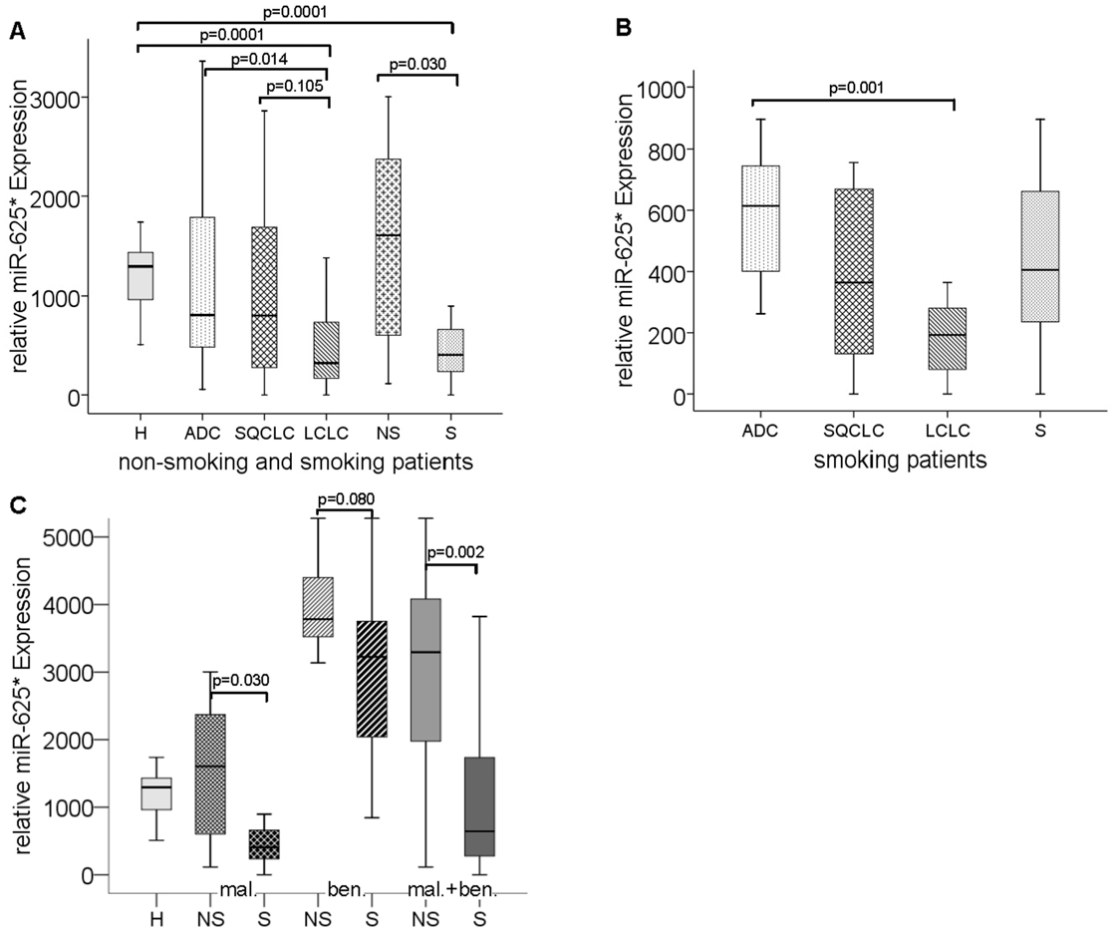
Correlation of serum levels of miR-625* with the histological type of carcinoma and smoking behavior. The box plots show the different, relative amounts of miR-625* in healthy controls with unknown smoking behavior (H, n = 30) and all NSCLC patients with ADC (n = 39), SQCLC (n = 35), LCLC (n = 12), and non-smoking (NS, n = 7) and smoking (S, n = 28) behavior (A), in smoking NSCLC patients (S, n = 28) with ADC (n = 12), SQCLC (n = 10) and LCLC (n = 6) (B), and in healthy controls (H, n = 30), NS (n = 7) and S (n = 28) with malignant lung tumors (mal.), NS (n = 9) and S (n = 11) with benign lung tumors (ben.), NS (n = 16) and S (n = 39) with malignant or benign lung tumors (mal.+ben.) (C). As determined by Mann and Whitney-U test, the significant p values of the statistical evaluations of serum RNA and miR levels are indicated above the blots.

Fig. 4A shows the prevalence of serum values of miR-625* in NSCLC patients with the different histological types, as well as in non-smoking and smoking NSCLC patients, whereas Fig. 4B only shows this prevalence in smoking NSCLC patients. As depicted in Fig. 4A, LCLC patients (p = 0.014) and smoking patients (p = 0.030) had significantly lower serum levels of miR-625* than ADC patients and non-smoking patients, respectively. Within the group of smoking patients, the LCLC patients had lower serum values of miR-625* (p = 0.001) compared to ADC patients (Fig. 4B). In the cohort of the patients with benign lung tumors the smoking patients also had lower levels of miR-625* than the non-smoking patients, but this difference was not significant (p = 0.080). However, when we merged both cohorts of NSCLC patients and patients with benign lung disease, we detected more significantly lower serum levels of miR-625* in smoking patients (p = 0.002) than non-smoking patients (Fig. 4C). Regrettably, the smoking behavior of healthy individuals was unknown to consider the relationship of the levels of miR-625* with the smoker status of healthy individuals.

Since there were only two cases with asbestos exposure in our patient cohort, we could not examine whether the decrease in miR-625* expression was associated with this carcinogen. Apart from these associations, no further significant correlation between clinical parameters and the serum values of miRs were detected.

### MiR-361-3p and miR-625* do not affect the protein expression of SMAD2 and TGF-ß1 in A549 cells

The cancer-specific decrease in transcript levels of miR-361-3p and miR-625* in NSCLC patients provoked us to search for potential targets of miR-361-3p and miR-625* in the miRbase database [16]. The screening revealed potential binding affinities of miR-361-3p to Smad2 and of miR-625* to TGFß1. To examine whether expressions of Smad2 was regulated by miR-361-3p and those of TGFß1 by miR-625*, we performed transfections of the cell line A549 using mimics and inhibitors of miR-361-3p and miR-625*. The mimics are double-stranded RNA molecules which mimics the mature of endogenous miR-361-3p and miR-625*, whereas the inhibitors are single-stranded, modified RNA molecules which after transfection, specifically binds to mimics and endogenous miR-361-3p and miR-625*, and inhibits their function. The data of the quantitative real-time PCR using gene-specific primers and Western blotting using antibodies specific for SMAD2 and TGF-ß1 showed no effect of the mimics and inhibitors on the RNA and protein levels of SMAD2 and TGF-ß1. These findings indicate that miR-361-3p and miR-625* do not target SMAD2 and TGF-ß1 (data not shown).

## Discussion

In the present study we performed microarray profiling of 1158 different miRs in serum of 21 NSCLC patients and 11 healthy individuals. Out of the 30 most dysregulated miRs of the array, we selected the two most significantly down-regulated miRs (miR-361-3p and miR-625*), which we validated further in serum of 97 NSCLC patients, 20 patients with benign lung disease and 30 healthy individuals by quantitative real-time PCR. Our data show that in contrast to the elevated serum levels of total RNA in NSCLC patients, the serum levels of both miRs decreased in NSCLC patients compared with the levels in patients with benign disease and healthy controls. Since these serum miR measurements could discriminate NSCLC patients from benign lung disease and healthy controls, their low serum levels may represent a cancer-specific dysregulation with potential functional consequences. This assumption is further supported by the observation that the low transcript levels increased back to the normal levels in NSCLC patients after surgical removal of their primary tumor. Furthermore, the decreased serum levels of miR-625* correlated with the LCLC subtype and the smoking behavior of NSCLC patients.

To date, no study has quantified the transcript levels of miR-361-3p and miR-625* in tumor tissue or blood. In our present study, these both miRs could differentiate NSCLC from benign lesions and healthy controls. Screening for their putative target mRNA molecules in the miR database (miRBase) showed that miR-361-3p may down-regulate the gene expression of paired-box (PAX) [17] and ras-related rap2 protein RAP2B which belongs to Ras superfamily of GTPases [18] by targeting the 3′-UTR of the corresponding mRNA molecules. As a result the down-regulation of miR-361-3p might lead to higher protein levels of PAX and RAP2B in NSCLC patients. PAX genes encode a family of paired-box transcription factors which are required for the growth and survival of cancer cells. Frequent PAX gene expression has been identified in several tumor cell lines and primary tumor tissues, amongst others lung cancer [17]. The preferred target mRNA molecules of miR-625* seem to be growth factor pleiotrophin (PTN) [19] and metalloproteinase with thrombospondin motifs-1 (ADAMTS-1) [20]. PTN is highly expressed in certain solid cancers and activates its cell surface receptors, regulating multiple functions including cell adhesion, cell migration, cell proliferation and cytoskeletal stability [19]. Overexpression of ADAMTS-1 promotes pulmonary metastasis of Lewis lung carcinoma cells and a proteinase-inactivated mutant of ADAMTS-1 inhibited their metastasis, indicating that the prometastatic activity of ADAMTS-1 requires its metalloproteinase activity [20]. These examples of the miR database might allude to the significant down-regulation of miR-361-3p and miR-625* and their role in NSCLC.

Due to the high proliferation and turnover rate of tumor cells in the primary tumor, it was surprising to detect reduced concentrations of circulating miR-361-3p and miR-625* in serum of our NSCLC patient cohort. Alike the elevated serum levels of total RNA induced by necrosis or apoptosis, one might expect that the increased cell death also results in increased release of miRs in these patients. We cannot explain this phenomenon and speculate that the stability of these particular miRs might be affected. Moreover, the low levels could also be caused by an altered enzymatic activity in blood or a modulated expression of the enzyme Dicer that is essential for the biogenesis of mature miRs [21], [22]. However, these effects might also influence the transcript level of other miRs. A further explanation could be that the primary tumor affects lymphocytes in a paracrine manner resulting in the reduced expression of miR-361-3p and miR-625*. Since no lymphocytes were available from our present patient cohort, we intend to investigate this hypothesis in the future using another NSCLC patient cohort with matched serum and lymphocyte samples.

Increased frequency of cigarette smoking is associated with lung cancer risk. Therefore, we also investigated the molecular relationship of cigarette smoking of lung cancer patients with the miR concentrations. Whereas all non-smoking patients had an ADC, the smoking patient subgroup had different histological types of ADC, SQCLC and LCLC. Statistical analysis showed that NSCLC patients with a LCLC and smoking patients had significantly lower serum levels of miR-625* than ADC and non-smoking patients, respectively. Within the subgroup of smoking patients, LCLC patients had the lowest miR-625* levels. No significant decrease was observed in non-smoking, ADC and SQCLC patients. Thus, our findings may support the notion that the dysregulation of serum miR-625* in smoking patients may be related to LCLC. In the cohort of the patients with benign lung tumors the smoking patients also had lower levels of miR-625* than the non-smoking patients, but this difference was not significant. However, when we merged both cohorts of patients with malignant and benign lung tumors, we detected more significantly lower serum levels of miR-625* in smoking patients than non-smoking patients. Although we do not know the smoking status of healthy individuals, these findings could allude to the tumor-specific (but not the cancer-specific) decrease in the serum levels of miR-625* in smoking patients. However, in our study the smoking status was only known of few patients. Therefore, further investigation is required using larger patient cohorts, and functional validation of miR-625* is planned to explore a causative relationship. In lung cancer tissues, the relationship with smoking status has also been described for other miRs [23], [24], [25]. Among NSCLC patients, low expression levels of miR-143 were significantly correlated with smoking behavior [24]. In individuals with a history of cigarette smoking, miR-218 expression was significantly reduced [23]. A link between miR-218 down-regulation and cigarette smoking was demonstrated by exposing human bronchial epithelial cells to cigarette smoke extract which decreased miR-218 expression levels [25].

As miRs are subject to complex regulatory mechanisms and bind to several different target mRNA molecules, we examined whether KRAS is a target of miR-625*. KRAS mutations were reported to be almost entirely limited to lung cancers in smokers [26]. However, screening of miR-625* in the miRbase database [16] revealed no binding, but binding affinity to TGFß1. As a multifunctional regulatory protein, TGFß1 controls many cellular functions, such as cellular proliferation, differentiation, migration, apoptosis, adhesion, angiogenesis, immune surveillance and survival. In carcinogenesis, TGFß1 has been suggested to play a dual role, acting as a tumor suppressor in early stages and a tumor promoter in later stages, by enhancing tumor cell motility, immunosuppression and invasiveness. Increased circulating levels of TGFß1 in blood may modulate cellular microenvironment and consequently lung cancer development and prognosis [27]. The intracellular signalling of TGFß1 is mediated by Smads. In the epithelial-mesenchymal transition (EMT) the increased transcriptional activity of phosphorylated Smad2 and 3 activates the expression of target genes, such as TGF-β1, MMP-2, MMP-9, plasminogen activator inhibitor type-1, vascular endothelial growth factor, Snail and Slug, thus promoting cancer cell mobility and invasion [28]. Of the numerous potential targets of miR-361-3p and miR-625*, we chose Smad2 and TGFß1 for our analyses. To examine whether Smad2 is regulated by miR-361-3p, and TGFß1 by miR-625*, we performed functional analyses. However, Western blot analyses showed no effect of 361-3p and miR-625* on Smad2 and TGFß1 protein expression in the lung cell line A549, respectively.

Interestingly, the differentially expressed miRs detected in our blood-based microarray profiling are different to those listed in previously published profiling studies [24], [29]. As far as we know, miR expression profiling in serum of lung cancer patients have only been carried out in three studies [30], [31], [32]. Foss et al. used a miR expression system containing 880 mature miRs and showed the utility of miR-1254 and miR-574-5p as serum-based biomarkers for minimally invasive screening [31]. One of the two other studies which applied Solexa sequencing showed that the signature of miR-486, miR-30d, miR-1 and miR-499 was an independent predictor of overall survival [32]. The other study showed that the elevated transcript levels of miR-25 and miR-223 in serum were blood-based biomarkers of NSCLC patients [30]. The detection of divergent miR signatures in microarray assays could mainly be due to the use of serum and the investigation of circulating miRs, but also to the different composition of the patient cohorts and the methodological differences used in the profiling studies. For example, microarrays can include precursor or mature miRs, while we used mature miRs for probe hybridization. Moreover, the number of miRs in our microfluid biochips was upgraded and contained 1158 different miRs, and therefore, more miRs than in arrays used in previous studies.

In our study we additionally quantified the miR levels in the serum of patients with benign lung disease, because on suspicion of a tumor it is important to distinguish between malignant and benign lesions. The comparison allows ascertaining that the dysregulation of serum miRs is cancer-specific and not only tumor-specific or the consequence of inflammation. Therefore, to accurately examine the pathology of a tumor in clinical practice, it is of high interest to establish minimal-invasive biomarkers able to discriminate a malignant from a benign tumor. To our knowledge, there are five publications dealing with circulating miRs in blood of NSCLC patients [30], [31], [32], [33], [34], and only one recent study compared the concentrations of serum miRs in lung cancer patients with those in patients with benign disease. The authors of that study developed a test, based on the detection of 34 serum miRs that could identify patients with early stage NSCLC in a population of asymptomatic high-risk individuals with 80% accuracy. The miR signature was able to distinguish between benign and malignant lesions, and to capture the onset of the malignant disease in individual patients over time [34].

In conclusion, we identified lung cancer-associated miRs by microarray profiling on microfluid biochips and quantitative real time PCR. Our results suggest a potential diagnostic relevance of miR-361-3p and miR-625* as blood-based markers. To further validate the clinical utility of these circulating miRs, a prospective large-scale study is planned. In addition, further functional analyses are intended, to characterize their cancer-specific role.
